# Depression, anxiety and its predictor among internally displaced person in metekel Ethiopia, 2023: using a structural equation model

**DOI:** 10.3389/fpsyt.2024.1458939

**Published:** 2025-01-22

**Authors:** Solomon Debela Bekeko, Teshome Demis Nimani, Samuel Demissie Darcho, Feyisa Shasho Bayisa

**Affiliations:** ^1^ Department of Public Health, Pawi Health Science College, Metekel, Ethiopia; ^2^ Department of Epidemiology and Biostatistics, School of Public Health College of Medicine and Health Science, Haramaya University, Harar, Ethiopia; ^3^ Department of Health Service and Policy Management, School of Public Health, College of Medicine and Health Science, Haramaya University, Harar, Ethiopia

**Keywords:** depression, anxiety, internally displaced person, and ethiopia, metekel zone

## Abstract

**Introduction:**

These individuals face psychological and physical trauma, loss of jobs, and emotional isolation, which may lead to the development of depression and anxiety. In 2022, 120 countries had over 71 million IDPs, a 20% increase from the previous year. In sub-Saharan Africa, natural disasters accounted for 40% of all new IDPs worldwide.

**Method:**

An institutional-based cross-sectional study was conducted in Ethiopia. A systematic random sampling method was used to select 997 respondents. Data were collected using a structured interview questionnaire. STATA Version 17 and Amos Version 21 were used for the analysis. Descriptive statistics were summarized using frequency, percentage, table, graph, chart, figure, and summary statistics. Structural equation modeling was employed to assess the relationship.

**Result:**

The overall prevalence of depression and anxiety was 79.64% (95% CI: 77.02 82.03) and 74.62% (95% CI =71.83%, 77.23%) respectively. age (adjusted *β* = 0.0034, 95% CI: 0.0012, 0.0056), history of the death of a loved one (adjusted *β* = 0.132, 95% CI: 0.0803, 0.185), had formal education (*β* = -0.164, 95% CI: -0.229, -0.098) occupation other (adjusted *β* = -0.183, 95% CI: -0.261, -0.105), Not having medically confirmed mental illness (adjusted *β* = -0.185, 95% CI: (-0.325, -0.045), PTSD score (adjusted *β* = 0.0082, 95% CI: 0.0048, 0.012) associated with anxiety.

**Conclusion:**

Displacement medically confirmed mental illness, death of a loved one, and post-traumatic stress disorder (PTSD) significantly impact anxiety. Policymakers should provide greater access to mental health management and prevention services, and medical practitioners should understand the connection between depression and anxiety.

## Introduction

Due to the devastation of homes, the environment, places of worship, political persecution, and economic necessity, conflict resulted in a large-scale displacement of people. Natural or man-made catastrophes have occasionally led to an increase in the global population of internally displaced people. People who are forced to leave their homes or regular places of residence due to armed conflict, widespread violence, human rights violations, or natural disasters are known as internally displaced persons, or IDPs (1). These people deal with a variety of issues, such as psychological and physical trauma, experiencing violence, losing their jobs, and being cut off from friends and family (2). All of these challenges may play a role in the development of post-traumatic stress disorder (PTSD), a prevalent mental health illness marked by hypervigilance, avoidance, nightmares, flashbacks, and emotional numbness (3).

Displacement is a stressful condition that disrupts families and has an impact on people’s physical and mental health at all ages. Millions of people have been displaced due to conflicts in at least two-thirds of Africa’s countries (4). Based on epidemiological studies, there is an increasing prevalence of mental health illnesses, especially among populations affected by and recovering from conflict (5). At the end of 2022, 120 countries worldwide had more than 71 million internally displaced persons as a result of violence, conflict, and natural catastrophes. This figure represents a 20% rise over the year before it (6). In sub-Saharan Africa, reports of human and natural disasters with the potential to cause IDPs have been widely reported (7). In 2020, 32 out of 54 African countries had natural disasters as their main cause of internal displacement, accounting for about 40% of all new internal displacements worldwide (7). The United Nations Human Rights Commission (UNHCR) reports that 42% of all internally displaced persons worldwide reside in Africa (8). Depending on the particular country and population under study, the prevalence of PTSD among internally displaced people (IDPs) varies greatly, from 3% to 88% (9).

In East Africa, the proportion of people with PTSD varies from 11% to 80.2% (10). According to a meta-analysis study carried out in sub-Saharan African nations, the prevalence of PTSD varies from 12.3% in Central Sudan to 85.5% in Nigeria, with most of them reporting having more than 50% of the prevalence (10). Studies on the prevalence of PTSD among internally displaced people (IDPs) from several African countries have also been carried out; the results show that southern Ethiopia (Gedeo 58.4%), Nigeria (42.2%), and Uganda (54%) have the highest rates (1).

There are several variables that have been linked to a higher incidence of PTSD in internally displaced people. These factors include the following: being a woman, being young, having experienced or witnessed violence, trauma, depression, anxiety, stress, having little education, having no social support, and facing economic challenges (11). In addition to the listed variables, there are several other factors that may raise the risk of PTSD in African IDPs. These variables include prolonged conflicts and instability in politics, which can exacerbate the trauma and displacement cycle and make it more difficult for internally displaced people to find safety and security. IDPs in Africa frequently experience poverty and food shortages, which add to their stress and hardships. These socioeconomic variables may exacerbate PTSD in these susceptible populations by creating emotions of helplessness, despair, and being trapped in challenging circumstances (12).

Moreover, studies on mental health problems (depression, and anxiety) among internally displaced people affected by conflict were examined using univariate analysis only, whereas more advanced models that take into account the complex relationships among mental health problems and risk factors are required. The interrelationships between mental illness and risk factors should be considered in the statistical analysis, and both direct and indirect paths should be investigated in the structural equation model.

Despite the high prevalence of depression and anxiety among IDPs in several Ethiopian regional states, there is no comprehensive research that demonstrates the prevalence of depression and anxiety and its associated factors. The objective of this study was to assess the prevalence of depression, anxiety, and its associated factors among internally displaced persons in Metekel, Ethiopia, in 2024.

## Method and materials

### Study setting, design, and period

This study was carried out in the Metekel zone, which is located in Benishangul Gumuz Regional State in North-West Ethiopia, in an area of IDP camps that has been devastated by violence. One of the three Benishangul Gumuz regional state administrative zones, the Metekel zone is located 338 kilometers from Assosa, a regional town, and 546 kilometers from Addis Ababa. The Amhara region borders the city of Gilgil Beles on the north and east, Kamashi on the south and southwest, and Sudan on the west. Gilgil Beles town serves as the administrative headquarters of the zone. As per the 2007 Census conducted by the Central Statistical Agency of Ethiopia (CSA), 276,367 people live in this zone, with 139,119 males and 137,248 women.

In 2019, the Metekel zone had an armed battle that became known as “conflict.” Many individuals were forced to leave the Metekel Zone as a result of the fighting there. At this point, over 7000 individuals still reside in IDP camps, even though the majority of the displaced people have returned and are living in post-community settings. In the Metekel Zone, there are four officially recognized internally displaced person sites: Dangur, Mandura, Debate, and Bullen sites, with 5080, 809, 1008, and 552 IDPs, respectively. There are currently 7,449 internally displaced people residing in these sites. An institutional-based cross-sectional study was conducted in the Metekel zone conflict-affected area in IDP Camps among internally displaced persons from April 2023, to June 2024.

### Population

Internally displaced people who resided in the Metekel Zone’s IDP camps in the conflict-affected area served as the study’s source population.

#### Inclusion criteria

All internally displaced people living in the four IDP camps in the Metekel zone who were at least eighteen years old were included in the study.

#### Exclusion criteria

Those under the age of eighteen who resided in the Metekel zone of the IDP sites, and who were critically ill and did not respond answer during data collection.

### Sample size determination and sampling procedure

**Figure 1:**
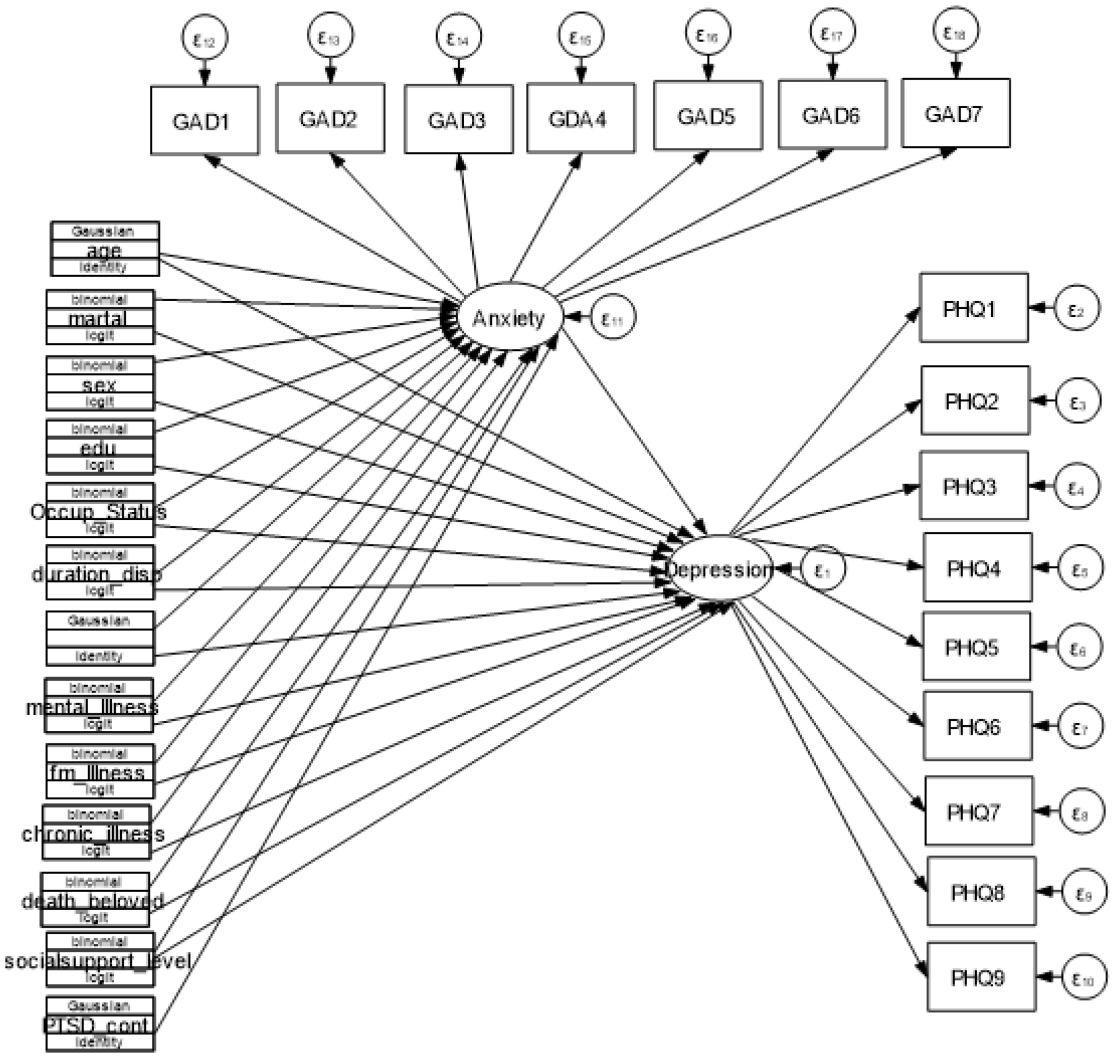
Hypothetical model for Depression Anxiety and determinants among IDPs in Metekel conflict area, 2023.

The sample size computation for structural equation modeling is based on the model’s complexity. When calculating the sample size for modeling structural equations, the general rule of thumb is to divide the number of model parameters that require statistical estimation (q) by the number of cases (N). This can vary from 5:20 times for each free parameter that needs to be estimated in the proposed model. Several sources suggest that, for the structural equation model (13). sample sizes of at least 150 are necessary (13). According to some scholars, at least a sample size of 200 to 500 should be used for structural equation modeling (14). For this study, a ratio of 13: 1 was used to obtain the minimum adequate sample size and address the objective. That means that for one free parameter in the hypothesized model, there should be 13 respondents. Based on the hypnotized model, there are a total of **73** free parameters to estimate (13 variances for exogenous variables, 26 regression coefficients, which are the coefficients between exogenous observed variables and latent variables, 1 regression coefficient between the latent variables, 1 covariance between the errors of latent variables, 14 loadings between latent variables and indicators except for those path coefficients fixed to one and 18 error variances for indicator variables) shown in **Figure 1**. By the thumb rule, it can be used in a ratio of 13:1, and the minimum sample size required is 949 (13 * 73). After adjusting for a 5% nonresponse rate, the total sample size for this study becomes 997. To select the sampling unit from the four IDPs, a Systematic random sampling technique was used. A sampling frame was created from the line list of all IDPs that was obtained from the Metekel Zone Disaster and Risk Management Office. Then by arranging the adult IDPs from 1 to N lists, the final sampling frame created for the study contained 5,016 from a total of 7,449 IDPs. The sampling interval (K) was calculated by dividing the sampling frame by the study sample size and it was 4.81 ≈ 5. Using a table of random numbers, we chose the first IDP from the sampling frame, and we continued choosing respondents based on the sampling interval or the additional participants were chosen at every K^th^ (5^th^) interval until the necessary sample size was reached.

### Study variables

#### Unobserved endogenous variables (outcome variables)

Depression and Anxiety

#### Observed exogenous variables (independent variables)

Socio-demographic factors: age, sex, marital status, educational status, and occupational status

Clinical relate factors: ever treatment for mental illness, family member treated for mental illness

Displacement factors: frequency of displacement, duration since the displacement

Relationship-related factors: death of a beloved one, social support

#### Operational definition

##### Depression

Based on a score of 10 or above on the Patient Health Questionnaire (PHQ-9) depression symptoms were declared to be present in a study participant. The total scores for the severity scale is 0–4 for no depression, 5–9 for mild depression, 10–14 for moderate depression, 15–19 for moderately severe depression, and 20–27 for severe depression (15).

##### Anxiety

Based on their overall score of 10 or above on the Generalized Anxiety Disorder-Seven (GAS-7), study participants were categorized as experiencing anxiety symptoms. When it comes to the severity scale, a total score of 4 or less denotes no anxiety, 5–9 mild symptoms, 10–14 moderate symptoms, and 15 or more denotes severe anxiety symptoms (16).

##### Social support

The study participants were divided into three social support categories based on their scores on the Oslo 3-item social support scale (OSS-3): weak social support (3–8), moderate social support (9–11), and strong social support (12–14, 17).

#### Data collection tools and procedure

An interviewer used a structured questionnaire that the investigator had created based on earlier research to gather primary data. Sociodemographic, clinical, displacement and relationship-related aspects were all included in the questionnaire. The questionnaire included individual items (indicators) measuring anxiety, depression, and post-traumatic stress disorder (20 items), with each item cross-referencing the latent variables (7 items). To ensure consistency, the data was collected by having an individual translate the English version into Amharic. The Patient Health Questionnaire 9 (PHQ–9) was used to assess depression and monitor its levels. The PHQ-9, with its distinct emphasis on the nine DSM-IV depressive disorder diagnostic criteria, is a widely used tool in primary healthcare settings. Nine items make up the Patient Health Questionnaire-nine (PHQ-9), with a total score range of 0 to 27. A score of 0 indicates “not at all,” a score of 1 indicates “several days,” a score of 2 indicates “more than half of the days,” and a score of 3 indicates “almost every day.” A score of 10 or higher is considered depressive. A sum of scores of 0–4 shows no depression, 5–9 suggests mild depression, 10–14 indicates moderate depression, and 15–19 indicates moderately severe depression. Scores can be translated to the severity measure (15, 18). The PHQ-9 has been validated in Ethiopia with the same culture, language and used among refugee-displaced persons (19). The reliability coefficient Cronbach’s was 0.89, showing good internal consistency (20).

Anxiety was assessed using Generalized Anxiety Disorder-Seven (GAD-7), which is a brief measure of anxiety that has seven items and is assessed on a four-point Likert scale with a total score range of 0 to 21, where 0 = “not at all,” 1 = “several days,” 2 = “more than half of the days,” and 3 = “almost every day.” Scores can be transferred into the severity measure, where a sum score of 4 or less is regarded as the absence of anxiety, 5–9 as a mild symptom, 10–14 as a moderate symptom, and 15 or more as a severe symptom of anxiety (16). The GAD-7 has adequate psychometric validity, including convergent validity, internal consistency, and test-retest reliability in various populations (16, 21).

#### Data processing and analysis

The collected data were coded and entered into Epi Data Version 4.6 and exported to STATA Version 17 and Amos Version 21 for further analysis. Descriptive analyses were done using texts, tables, graphs, charts, and figures for data summarization. Since the variables in this study were skewed (not normally distributed), the mean is biased by the values far end of the distributions. The reliability of the tools for depression and anxiety was assessed for each construct, depression, and anxiety for the current study using Cronbach’s alpha reliability coefficients. If Cronbach’s alpha coefficients are greater than 0.7, it is considered satisfactory. Individual items (indicators) cross-referencing the latent variables were used in the questionnaire, including depression (9 items), and anxiety (7 items), missing variables at random that are continuous and normally distributed amputated means and those categorical variable was amputated by multiple imputation.

#### Structural equation modeling

Structural Equation Modeling (SEM) was used to assess the complex relationship between different latent and observable variables. Structural equation modeling comprises two interrelated components: the measurement model and the structural model. The measurement model is a confirmatory factor analysis model used for grouping multiple indicators into some constructs, while the structural model (theoretical model) is used to evaluate the interrelationships between latent variables and the relationship between latent variables and observable predictors (22).

The advantages of SEM over other regression analyses are modeling measurement errors, simultaneously testing relationships, and best-fitting model and theory developments. The goals of SEM are to ascertain whether the data gathered are consistent with a theoretical model and to examine the proposed direct relationship between independent (exogenous) and dependent (endogenous) variables. Multiple variance and regression analyses are performed simultaneously as part of the SEM hypothesis testing process (23).

In this study, depression, and anxiety are latent variables that constitute items that measure them indirectly. The parameters of the population covariance matrix and sample covariance were compared by SEM analysis to assess model fit and estimate parameters. Moreover, standard error and individual parameter significance checks were carried out. To compare the models, the comparative fit index (CFI) was used for each model fitted and root mean square error of approximation (RMSEA).

#### Data quality control

Data was gathered using the structured questionnaire under the guidance and supervision of experienced and trained data collectors and supervisors. Supervisors received training before data collection to understand the questionnaire. Data collectors also received a one-day training on the objective of the study, the techniques of data collection, the content of the questionnaire, and the concern of confidentiality. Four trained BSc public health professionals and two Heath extension workers who were native in the local languages. The data collectors were supervised by trained BSc psychiatrists from Pawi General Hospital during the data collection process. Any ambiguity was resolved through communication with the supervisors. Furthermore, the principal investigator followed the entire data collection process Depression, and anxiety scales have been adopted and used many times in the Ethiopian context in post-conflict affected areas, among internally displaced persons and refugees (11, 12, 21, 24). Before data collection questionnaires were pre-tested in Assosa Zone Bambasi IDP camp talking 50 (5%) of the total sample size who were not included in the study.

### Ethical consideration

Ethical approval was obtained from the Institutional Review Board (IRB) of the University of Gondar, University of Medicine and Health Science, Institute of Public Health. The ethical clearance letter was submitted to the Metekel Zone Disaster and Risk Management Office for the care of IDPs. After receiving the approval and a letter of support from the zone, written informed consent was obtained from the participants. Data collection began after the purpose of the study, confidentiality, and its potential benefits were explained to the participants.

## Result

### Socio-demographic characteristics

The questionnaire was filled out by 997 randomly chosen people, yielding a 100 percent response rate. Female participants made up about 56.37a percent of the study’s total. With an average age of 45.39 and an SD of 12.73. Those who were married were 92.08 percent and those who were not literate 58.68 percent. Of those surveyed, farmers made up the majority (81.95%). Regarding the length of the displacement, over half (59.45 percent) of the respondents were relocated just once, while the majority (96.20 percent) of the displaced people lived in the camp for more than a year (96.49%). 99.2 percent had little social support **Table 1**.

**Table 1:** Socio-demographic characteristics of IDP respondents in Metekel Zone, Ethiopia, 2023 (n =997).

Variable	Categories	Frequency	Percent
Sex	Male Female	435 526	43.63 56.37
Marital status	Single Married	79 918	7.92 92.08
Educational status	No formal education Had formal education	585 412	58.68 41.32
Occupational status	Farmer Other	817 180	81.95 18.05
Duration of displacement	< 12 month > 12month	35 962	3.51 96.49
Frequency of displacement	One time Two times	592 405	59.38 40.62
Mental illness	No Yes	978 19	98.09 1.91
Family ill with a mental case	No Yes	963 34	96.59 3.41
Chronic illness	No Yes	764 233	76.63 23.37
Death of beloved	No Yes	750 247	75.23 24.77
Social support	Poor social support Moderate social support	989 8	99.2 0.8
PTSD	Mean + SD 39.35 + 9.91		
Age	45.39 + 12.73		

#### Proportion of anxiety

**Figure 2:**
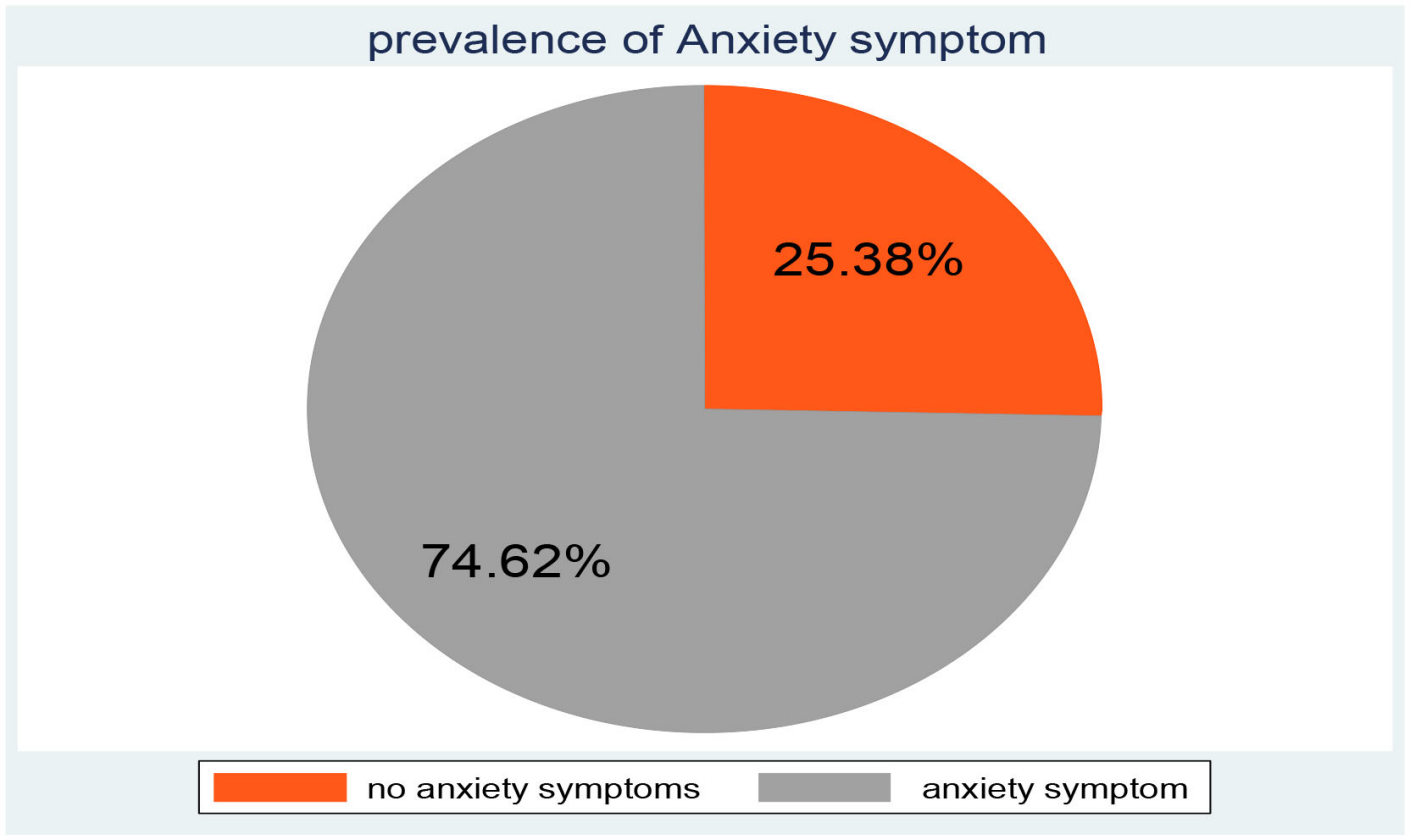
Proportion of anxiety among IDPs in Metekel Ethiopia 2023 (n =997).

In this study, the overall magnitude of anxiety was 74.62% (95% CI =71.83%, 77.23%) shown in **Figure 2**.

#### Proportion of depression

**Figure 3:**
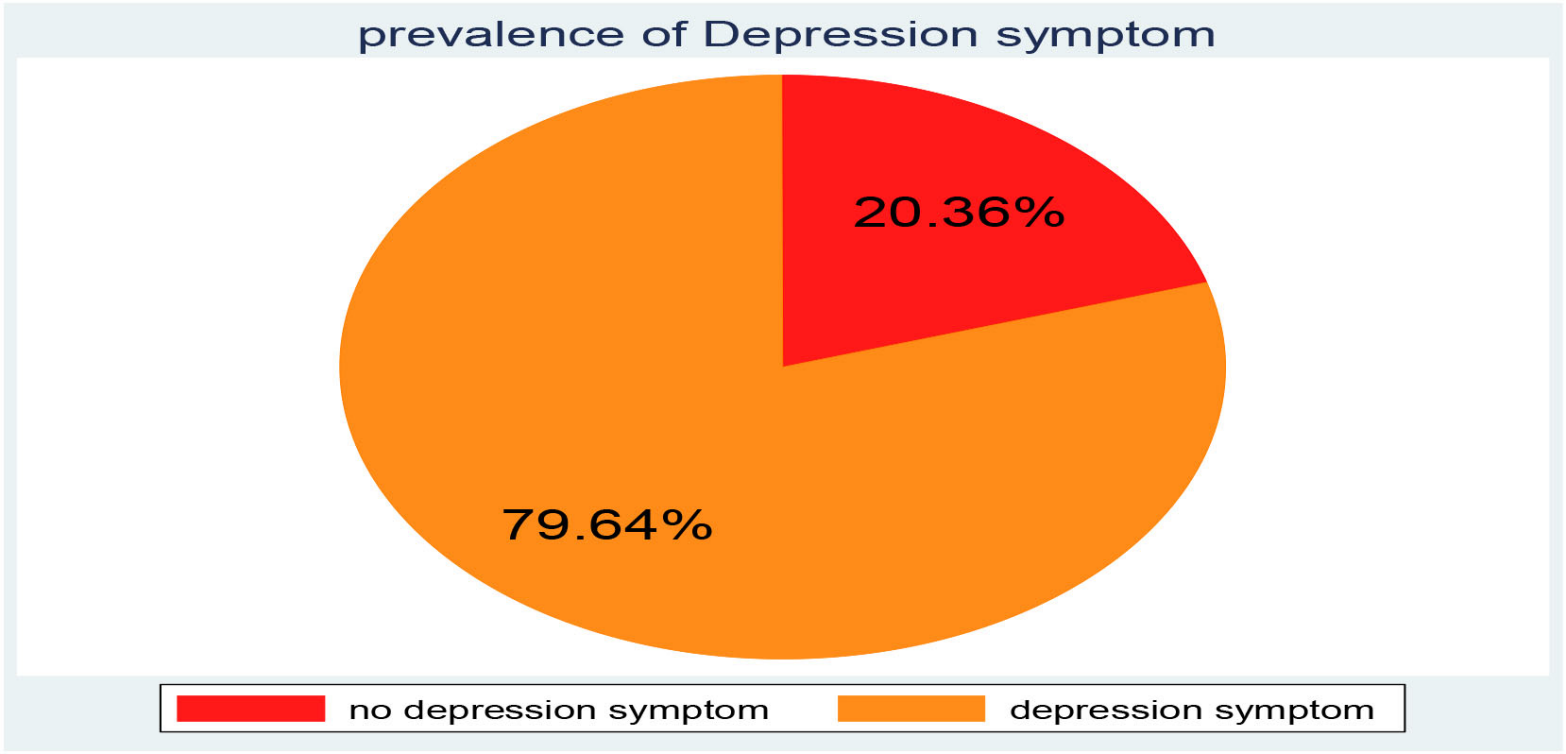
Proportion of depression among IDPs in Metekel Ethiopia 2023 (n =997).

In this study, the overall magnitude of depression was 79.64% (95% CI: 77.02 82.03) show in **Figure 3**.

### Factor associated with depression, among internally displaced persons in metekel Ethiopia

**Figure 4:**
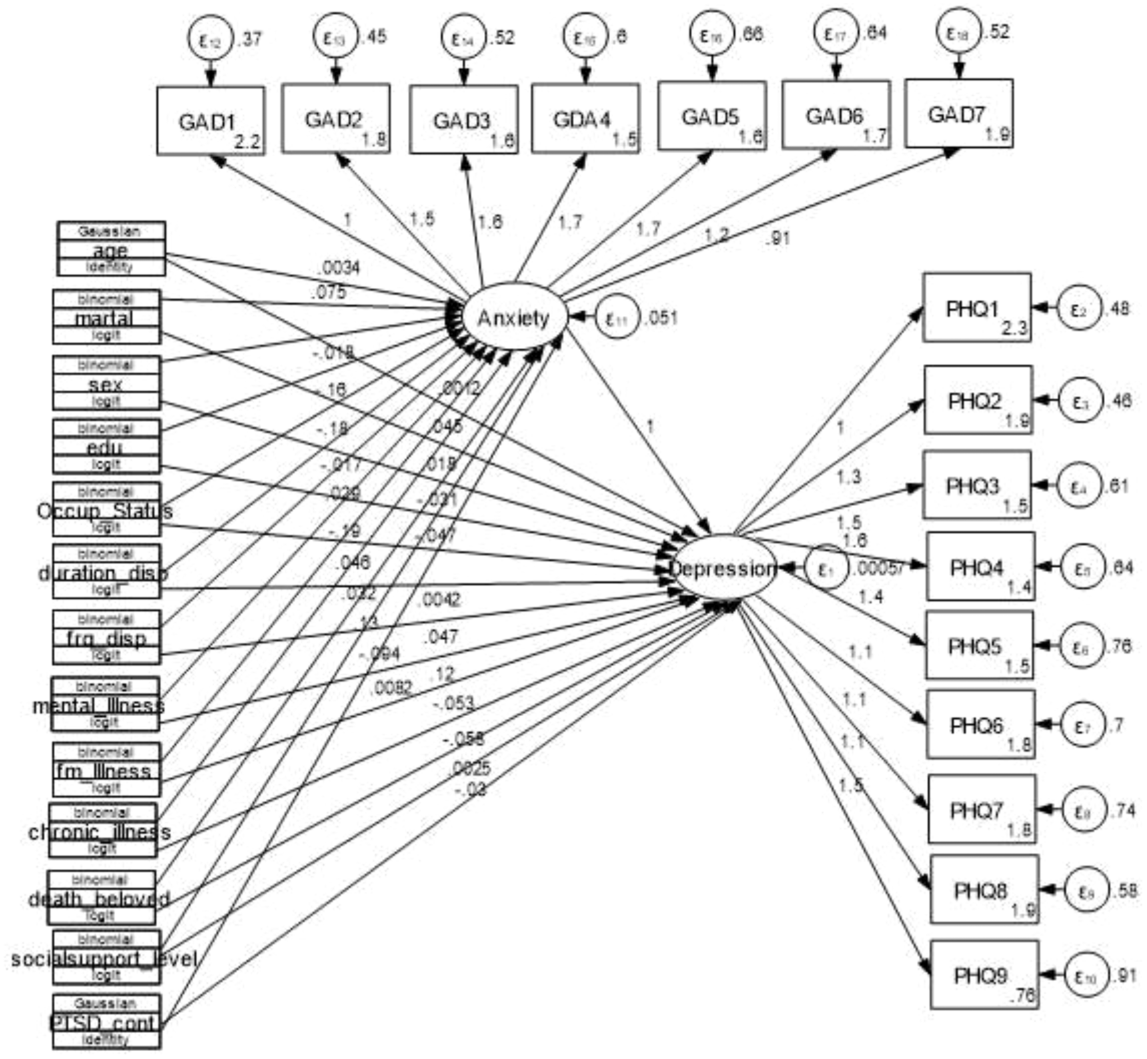
the final structural equation model showing the association between predictors and outcomes and between anxiety and depression among conflict-affected internally displaced persons in Metekel Ethiopia, 2023.

From the final SEM, factors of frequency of displacement, medically confirmed mental illness, and anxiety were significantly related to depression. However frequency of displacement, having medically confirmed chronic illness and having anxiety were positive relation with the mean score of depression. Having no medically confirmed chronic illness the mean score of depression was decreased by 0.057 (adjusted β= -0.057, 95% CI: -0.106, -0.0089) compared with those who had medically confirmed chronic illness. Exposed to the frequency of internal displacement was two times the mean score of depression was increased by 0.047 (adjusted β= 0.047, 95% CI: 0.0046, 0.089) compared to one-time displaced and the mean score of anxiety significantly increased depression by 1.025 (adjusted β= 1.025, 95% CI: 0.72, 1.319) by keeping other factor constants (**Table 2**, **Figure 4**).

**Table 2:** Factor associated with depression among internally displaced persons in metekel Ethiopia, 2023 (n =997).

Variable DV: Depression	Categories	Direct effect β (95% CI)	Indirect effect β (95% CI)	Total effect β
Sex	Male Female	1 0.018 (-0.019, 0.056)	1 0.018 (-0.019, 0.056)	1 0.036
Marital status	Single Married	1 0.045 (-0.041, 0.131)	1 0.045 (-0.041, 0.131)	1 0.09
Educational status	No formal education Had formal education	1 -0.031 (-0.082, 0.021)	1 -0.031 (-0.082, 0.021)	1 -0.062
Occupational status	Farmer Other	1 -0.046 (-0.121, 0.027)	1 -0.046 (-0.121, 0.027)	1 -0.092
Duration of displacement	< 12 month > 12month	1 0.0042 (-0.099, 0.107)	1 0.0042 (-0.099, 0.107)	1 0.0084
Frequency of displacement	One time Two times	1 0.047 (0.0046, 0.089)**	1 0.047 (0.0046, 0.089)	1 0.094
Mental illness	No Yes	1 0.116 (-0.018, 0.0205)	1 0.116 (-0.018, 0.0205)	1 0.232
Family ill with a mental case	No Yes	1 -0.053 (-0.154, 0.048)	1 -0.053 (-0.154, 0.048)	1 -0.106
Chronic illness	No Yes	-0.057 (-0.106, -0.0089)** 1	-0.057 (-0.106, -0.0089) 1	-0.057 1
Death of beloved	No Yes	1 0.0024 (-0.046, 0.0512)	1 0.0024 (-0.046, 0.0512)	1 0.0024
Social support	Poor social support Moderate social support	1 -0.0302 (-0.267, 0.207)	1 -0.0302 (-0.267, 0.207)	1 -0.0302
Age		0.0012 (-0.0011, 0.0034)	0.0012 (-0.0011, 0.0034)	0.0024
Anxiety		1.025 (0.729, 1.319)**		2.05

** Show that statistically significant at 5% and 1% level of significance.

### Factor associated with Anxiety among internally displaced persons in metekel Ethiopia

Factors such as age, educational status, occupational status, medically confirmed mental illness, death of a beloved one, and PTSD had statistically significant impacts on anxiety. Age, death of a beloved one, and PTSD were positive relationships with Anxiety, whereas having no educational status, and having no medically confirmed mental illness.were negative relationship with anxiety.

The age of the respondent increased by one year also the score of anxiety also increased by 0.0034 (adjusted *β* = 0.0034, 95% CI: 0.0012, 0.0056), While having a history of the death of a loved one during the displacement the score of anxiety increased by 0.132 (adjusted *β* = 0.132, 95% CI: 0.0803, 0.185) compared with those who had no history of the death of a loved one during the displacement, For participants who had formal education, anxiety scores decreased by 0.164 (*β* = -0.164, 95% CI: -0.229, -0.098) compared to those who had no formal education, Being the occupation of other the score of anxiety decreased by 0.183 (adjusted *β* = -0.183, 95% CI: -0.261, -0.105) compared to the farmer, Not having medically confirmed mental illness the score of anxiety was decreased by 0.185 (adjusted *β* = -0.185, 95% CI: (-0.325, -0.045) compared to that medically confirmed mental illness, and When the score of PTSD increases also anxiety increases by 0.0082 (adjusted *β* = 0.0082, 95% CI: 0.0048, 0.012) (**Table 3**, **Figure 4**).

**Table 3:** Factors associated with anxiety among internally displaced persons in metekel Ethiopia, 2023 (n =997).

Variable DV: Anxiety	Categories	Direct effect β (95% CI)	Indirect effect	Total effect β (95% CI)
Sex	Male Female	1 -0.018 (-0.058, 0.022)	—————	1 -0.018 (-0.058, 0.022)
Marital status	Single Married	1 0.075 (-0.019, 0.169)	—————	1 0.075 (-0.019, 0.169)
Educational status	No formal education Had formal education	1 -0.164 (-0.229, -0.098)**	—————	1 -0.164 (-0.229, -0.098)**
Occupational status	Farmer Other	1 -0.183 (-0.261, -0.105)**	—————	1 -0.183 (-0.261, -0.105)**
Duration of displacement	< 12 month > 12month	1 -0.017 (-0.124, 0.089)	—————	1 -0.017 (-0.124, 0.089)
Frequency of displacement	One time Two times	1 0.028 (-0.014, 0.0786)	—————	1 0.028 (-0.014, 0.0786)
Mental illness	No Yes	-0.185 (-0.325, -0.045)** 1	—————	-0.185 (-0.325, -0.045)** 1
Family ill with a mental case	No Yes	1 0.045 (-0.058, 0.149)	—————	1 0.045 (-0.058, 0.149)
Chronic illness	No Yes	1 0.0318 (-0.014, 0.0786)	—————	1 0.0318 (-0.014, 0.0786)
Death of beloved	No Yes	1 0.132 (0.0803, 0.185)**	—————	1 0.132 (0.0803, 0.185)**
Social support	Poor social support Moderate social support	1 -0.094 (-0.366, 0.178)	—————	1 -0.094 (-0.366, 0.178)
Age		0.0034 (0.0012, 0.0056)**	—————	0.0034 (0.0012, 0.0056)**
PTSD		0.0082 (0.0048,0.012)**	—————	0.0082 (0.0048,0.012)**

** Show that statistically significant at 5% and 1% level of significance.

## Discussion

This study revealed that the overall prevalence of depression among internally displaced persons was 79.64% (77.02, 82.03). The finding of this study was higher than the study in Uganda (30.7%) (25), South Sudan (50%) (26), Somali (59%) (27), Southeast Ethiopia (38.3%) (28), Eritrean Refugees (37.8%) (29), a pooled prevalence of 81 studies done using a systematic study (26.4%) (30), Mozambique (64%) (31), Uganda (58%) (32), southern Ethiopia (53.3%) (33), northern Ethiopia (39.3%) (34), Sweden (40.2%) (35), Sri Lanka (22.2%) (36), and Somalia (32.1%) (37). The possible justification may be compared to Ethiopia, where countries with high incomes offer refugees better psychosocial and financial supports that may be protective against mental diseases.

However, the findings of this study were lower than those in Tigray, northern Ethiopia (81.2%) (38), and Bangladesh (89%) (39). The variation may have resulted from the use of different assessment tools in each study: researchers used the Harvard Trauma Questionnaire in Uganda, the Traumatic Stress Symptom Scale in Turkey, and the Screen for Posttraumatic Stress Symptoms in Syria. Another reason may be that the studies were carried out among different populations in different cultural and social contexts to manage displacement problems.

The current study revealed that the prevalence of anxiety among internally displaced persons was 74.62% (71.83, 77.23). The findings of this study were higher than those in Mozambique (40%) (31), southwestern Uganda (73%) (32), northeast Ethiopia (33.4%) (24), Sweden (31.8%) (35), Somalia (34.9%) (37), and Sri Lanka (32.6%) (36). Since what is considered trauma in one culture might not be considered trauma in another, the characteristics of particular populations and their cultures can have an impact on the prevalence of psychiatric morbidity (40). Another reason might be the type and severity of the psycho-trauma that was experienced. The greatest risk factors for PTSD include intimate sexual violence and interpersonal violent trauma (41).

However, the findings of this study were lower than those in Nepal (80%) (42) and Afghanistan (84.6%) (43). The differences may stem from the diverse screening methods employed in other nations. A study conducted in the Gedo zone utilized the Hospital Anxiety and Depression Subscale (HADSS); a study conducted in Columbia used the Zung Anxiety Scale; studies conducted in Nepal and Afghanistan used the Hopkins Symptom Checklist-25 to screen for anxiety symptoms. Variations might also exist depending on the cutoff point that is chosen for every tool.

Frequency of displacement medically confirmed mental illness, and anxiety were significantly related to depression, and age, educational status, occupational status, medically confirmed mental illness, death of a loved one, and PTSD had statistically significant impacts on anxiety.

A study showed that the death of a loved one significant impact supported by a study conducted in Nigeria (44), and Nazon (45). Age study conducted on Internally Displaced Persons and Returnees in Georgia (46). A PTSD symptom score and having formal education study were incorporated on Colombians internally displaced by armed conflict (47), Among Internal Displaced People in South Ethiopia (11). The findings also showed that people who had lost family members through internal displacement had raised the score of anxiety. This is supported by the study done in Nigeria (48). This could be explained by the fact that the effects of losing a loved one can be similar to those of trauma victims who have gone through other kinds of trauma. These effects include intrusive negative thoughts like thoughts of retaliation, reminders of the event, and a possible significant impact on mental health (49).

The results of the study show that patients with anxiety symptoms were increased by 1.025 in depressed individuals. This study is supported by the study conducted in Mozambique (31) and in Kenya (50). Another statistically significant factor for depression was the frequency of shifting more than twice. As the frequency of postponement increased, the number of participants who were depressed increased by 0.047. This study is consistent with the study conducted in Ethiopia (51), Somalia (52), and systematic review in Africa (51). In this study, participants showed chronic illness was statistically significant. For participants who had no medically confirmed chronic illness, the mean score of depression was decreased by 0.057. This finding is supported study done in Lebanon (53, 54).

The concurrent evaluation of the direct and indirect effects of several independent variables on dependent variables of anxiety and depression was investigated as the study’s strongest point. The study was not without limits, though. The fact that this study only included adult internally displaced individuals may have an impact on the study’s generalizability to all internally displaced individuals and recall bias occurred.

## Conclusion

The prevalence of depression and anxiety among internally displaced persons was 79.64%, and 74.62% respectively. Frequency of displacement medically confirmed mental illness, and anxiety were significantly related to depression, and age, educational status, occupational status, medically confirmed mental illness, death of a loved one, and PTSD had statistically significant impacts on anxiety.

For Zonal disaster and risk management office: Screening services for mental health disorders should be needed and properly implemented, especially for females in the IDP camps. IDPs would be assisted in returning to their homes, supporting a healing process, and assisting in the rebuilding of their lives if the conflict could be resolved in a meaningful way. For health professionals: It is important to realize the interrelationship between anxiety, posttraumatic stress disorder, and depression. Thus mental illness (anxiety, posttraumatic stress disorder, and depression) should be suspected in IDPs who were displaced due to conflict. For internally displaced persons: It is better to seek medical help if symptoms of anxiety, posttraumatic stress disorder, and depression happen. It is preferable to have close relationships with other friends.

Studies on out of internally displaced persons are recommended. Medical practitioners must understand the connection between depression and anxiety. Internally displaced individuals should seek medical attention if they experience signs of depression or anxiety. Research on a mixed approach to internally displaced people is advised for researchers.

## Data Availability

The raw data supporting the conclusions of this article will be made available by the authors, without undue reservation.
